# The Japanese version of the National Cancer Institute’s patient-reported outcomes version of the common terminology criteria for adverse events (PRO-CTCAE): psychometric validation and discordance between clinician and patient assessments of adverse events

**DOI:** 10.1186/s41687-017-0022-5

**Published:** 2018-01-05

**Authors:** Takashi Kawaguchi, Kanako Azuma, Motohiko Sano, Soan Kim, Yosuke Kawahara, Yoko Sano, Tomohide Shimodaira, Keiichiro Ishibashi, Tempei Miyaji, Ethan Basch, Takuhiro Yamaguchi

**Affiliations:** 1 Department of Practical Pharmacy, School of Pharmacy, Tokyo University of Pharmacy and Life Sciences, 1432-1, Horinouchi, Hachioji, Tokyo, Japan; 2 Department of Pharmacy, Tokyo Medical University Hospital, 6-7-1, Nishishinjuku, Shinjuku-ku, Tokyo, Japan; 3 Department of Pharmacy Services, Saitama Medical Center, Saitama Medical University, 1981, Kamoda, Kawagoe-city, Saitama, Japan; 4 Department of Pharmacy, Juntendo University Nerima Hospital, 3-1-10, Takanodai, Nerima-ku, Tokyo, Japan; 5 Department of Pharmacy, Toshiba General Hospital, 6-3-22, Higashioi, Shinagawa-ku, Tokyo, Japan; 6 Department of Digestive Tract and General Surgery, Saitama Medical Center, Saitama Medical University, 1981, Kamoda, Kawagoe-city, Saitama, Japan; 7 Department of Clinical Trial Data Management, Graduate School of Medicine, The University of Tokyo, 7-3-1, Hongo, Bunkyo-ku, Tokyo, Japan; 8 Division of Health Care Research, QOL Research Group, Center for Public Health Sciences, National Cancer Center, 5-1-1 Tsukiji, Tokyo, Japan; 9 Department of Medicine, University of North Carolina, Chapel Hill, NC USA; 10 Division of Biostatistics, Tohoku University Graduate School of Medicine, 1-1, Seiryo-machi, Aoba-ku, Sendai, Miyagi Japan

## Abstract

**Background:**

The Patient-Reported Outcomes version of the Common Terminology Criteria for Adverse Events (PRO-CTCAE) was developed by the National Cancer Institute as an adverse event assessment system to evaluate patients’ symptoms, which tend to be underestimated in cancer clinical trials. The aim of this study was to assess the psychometric properties of the Japanese version of the PRO-CTCAE and the degree of adverse event assessment discordance between clinicians and patients.

**Methods:**

A total of 187 cancer patients receiving systemic therapy were enrolled. Reproducibility, criterion validity, and responsiveness of the Japanese version of PROCTCAE were assessed. The EORTC QLQ-C30 was used as an external anchor. Discordance of assessment of adverse events between clinician and patients were also assessed using the CTCAE and PRO-CTCAE.

**Results:**

A total of 187 participants (187 for criterion validity, 80 for reproducibility, and 100 for responsiveness), were analyzed (Mage = 62.4 years). All patients responded to at least one symptom item (M = 16). The mean (SD) intra-class correlation coefficients of overall reproducibility for the Japanese PRO-CTCAE was 0.63 (0.02). The correlation coefficient for the corresponding items in the EORTC QLQ-C30 and the Japanese PRO-CTCAE was high (Pearson *r* = 0.56–0.76). The analysis of responsiveness revealed significant dose-response trends (Jonckheere-Terpstra test, ps < 0.001). Depending on the adverse events, a discrepancy was observed in evaluation between the clinician and patient.

**Conclusions:**

These results revealed that there is underestimation in the assessment of adverse events in Japan, and that the Japanese version of the PRO-CTCAE had acceptable reliability and validity for common and clinically important symptoms.

## Background

The National Cancer Institute’s (NCI’s) Common Terminology Criteria for Adverse Events (CTCAE) is the current standard and predominant system for describing the severity of adverse events and is used worldwide, especially in cancer clinical trials [1, 2]. The Cancer Therapy Evaluation Program (CTEP) developed and released the original Common Toxicity Criteria in 1984, and the NCI’s CTCAE 4.0, distributed in 2009, is the latest version of the document. It is composed of 790 items and is harmonized with terminology used in the Medical Dictionary for Regulatory Activities (MedDRA). Although the CTCAE was developed by clinical experts, and has been the de facto standard method to report adverse events, its clinical validity has not yet been proven methodologically. Adverse events in the CTCAE, which can be classified into three general categories based on laboratory reports, clinical observation, and symptoms, are reported by a clinician; however, disagreement between clinicians and patients regarding symptom assessment has been revealed in research using a quality of life questionnaire (the European Organisation of Research and Treatment of Cancer Quality of Life Questionnaire Core 30, EORTC QLQ-C30), and in research on adverse event reporting using the modified CTCAE, which can use patients as respondents [3–13]. This evidence demonstrates that clinicians tend to underestimate the incidence and severity of symptoms in comparison with that reported by patients (i.e., patient-reported outcomes, PRO).

There is growing awareness of collecting symptom data using PRO; therefore, the NCI developed the “Patient-Reported Outcomes version of the Common Terminology Criteria for Adverse Events (PRO-CTCAE)” instrument to assess adverse symptomatic events that occur in clinical trials directly from patients’ responses. Of the 790 adverse events included in the CTCAE, 78 symptoms were identified as amenable to patient self-reporting, and the technical language was changed into plain expression [14]. After evaluating content validity through a cognitive interview study, a multicenter study was conducted to examine the construct validity, reliability, and responsiveness of the instrument [15, 16]. This validation study included 940 patients with various types of cancer, out of which 522 (55.5%) had received chemotherapy in the 2 weeks preceding data collection, and 161 (17.1%) were a 2 to 4 on the Eastern Cooperative Oncology Group Performance Status (ECOG PS) scale. The results of this validation study showed favorable psychometric properties in diverse participants.

Various language versions of the PRO-CTCAE (e.g., German, Danish, and Spanish) have been developed [17–19]. In addition to these, a Japanese version of the PRO-CTCAE has also been developed and linguistically validated [20]. The aim of the present study was twofold. First, we aimed to reveal discordance in symptom assessment between clinicians and Japanese patients with cancer. Second, we sought to examine the psychometric properties of the Japanese version of the PRO-CTCAE, including its construct validity, reliability, and responsiveness, and to confirm that the results were not significantly different from the results of the original validation study [16].

## Methods

### Participants

Patients initiating chemotherapy at four hospitals, namely Tokyo Medical University Hospital, Saitama Medical University Saitama Medical Center, Juntendo University Nerima Hospital, and Toshiba General Hospital, were invited to participate. Patients who were over 20 years of age, with any verified cancer, currently receiving systemic therapy for cancer, and with any score on the ECOG PS were eligible. All patients were required to possess sufficient Japanese language ability to understand and complete the questionnaire without assistance. Patients with cognitive impairment or any severe psychiatric disorder were excluded.

### Measurement

**The Japanese version of the PRO-CTCAE**. The original version of the PRO-CTCAE comprises 124 self-administered items, reflecting 78 symptomatic adverse events, and consists of five dimensions (presence, amount, frequency, severity, and interference with daily activities). Among these dimensions, one to three dimensions are assigned per symptom, which were selected based on attributes included in the original CTCAE items and the nature of each symptom. The recall period is the last 7 days. The psychometric properties were investigated by Dueck and colleagues, including assessment of construct validity, test-retest reliability, and responsiveness [16]. The Japanese version of the PRO-CTCAE has been developed and linguistically validated [20]. To limit burden on participants, only 39 items and 20 corresponding symptoms were used in the present study (see Appendix 1). These choices were based on research that has identified core symptoms that are common and clinically important to measure in clinical oncology trials, and were also used as “core symptomatic adverse events” in the development of the original version of the PRO-CTCAE [16, 21]. The following symptoms were selected: “anxiety;” “constipation;” “decreased appetite;” “dry mouth;” “fatigue, tiredness, or lack of energy;” “insomnia (including difficulty in falling asleep, staying asleep, or waking up early);” “loose or watery stools (diarrhea);” “mouth or throat sores;” “nausea;” “numbness or tingling in hands or feet;” “pain;” “sad or unhappy feelings;” “shortness of breath;” “vomiting;” “swelling in arms or legs;” “hair loss;” “headache;” “problems with concentration;” “problems with tasting food or drink;” and “appearance of a rash.” Assessment of adverse events related to these symptoms was based on their presence or absence, and three attributes: frequency, severity, and/or interference with daily life. The following were the response items for each attribute: Frequency: “*Never/Rarely/Occasionally/Frequently/Almost constantly/Not applicable,*” Severity: “*None/Mild/Moderate/Severe/Very severe*,” Interference with daily activities and amount: “*Not at all A little bit/Somewhat/Quite a bit/Very much,*” and Presence “*Yes/No*.”

### Anchor

As external information to assess the validity and responsiveness of the Japanese version of the PRO-CTCAE, the ECOS PS, Karnofsky Performance Status (KPS), CTCAE, EORTC QLQ-C30, and clinical global impression of change (GIC) were assessed. The KPS and ECOG PS are widely used to assess the functional status of cancer patients and the predictability of health outcomes [22–27]. Scores on the ECOG PS range from 0 to 5, representing “*fully active, able to carry out all pre-disease performance without restriction*” to “*dead*.” The Japanese version of the ECOG PS is a 5-item version developed by the Japan Cooperative Oncology Group (JCOG). Items are rated from 0 to 4, and exclude the rating of 5 (*dead*). The KPS consists of 11 categories, with scores ranging from 0 to 100, representing “*dead*” to “*normal, no complaints; no evidence of disease*.” In daily practice, the severity of adverse events is assessed by clinicians using the NCI-CTCAE version 4.0, distributed by JCOG, and the rating is documented in patients’ medical records. From these clinician-reported adverse events, we collected 20 adverse events corresponding to items on the Japanese version of the PRO-CTCAE used in this study. The EORTC QLQ-C30 is used to assess health related quality of life and is composed of five functional statuses, three symptoms, and six individual items. As with the performance status, some domains (e.g., physical function, pain, and appetite loss) of the EORTC QLQ-C30 are suggested to provide prognostic information [28]. The Japanese version of the EORTC QLQ-C30 has been validated in patients with cancer [29]. Each item is scored on a 4-point Likert-type scale, except for two items in the domain of global health status/quality of life (GHS/QOL), which uses a 7-point scale. All scores were linearly transformed to a 0 to 100 scale. A higher function score and lower symptom score represents better status. The GIC used in this study is a 7-point scale in which −3 = *much worse*, −2 = *moderately worse*, −1 = *a little worse*, 0 = *almost the same*, 1 = *a little better*, 2 = *moderately better*, and 3 = *much better*. Patients completed the GIC during Visit 2. The responses on the GIC were grouped as follows: items −3 to −1: *worse*, item 0: *almost the same*, and items 1 to 3: *better*.

### Demographics

Demographic information, including age, gender, type of cancer, and type of therapy, were obtained from medical records. Marital status, education level, employment status, and type of household were collected from the patient using a questionnaire.

### Study design

Participants were registered in one of two cohorts, based on their chemotherapy schedule, to avoid an extra clinic visit. The first group, Cohort A, was asked to complete the questionnaire on consecutive days in order to assess the reproducibility of the Japanese version of the PRO-CTCAE. Participants assigned to the second group, Cohort B, were used to assess responsiveness between Visit 1 and Visit 2. The interval between these visits was defined as 7 days, based on the recall period of the PRO-CTCAE in this study; however, a delay of 3 days was acceptable for Visit 2. Additionally, their clinicians rated PS and CTCAE during Visit 1. For both cohorts, patients were asked to complete the Japanese version of the PRO-CTCAE and EORTC-QLQ-C30 on Visit 1. Furthermore, patients in Cohort B were asked to rate the GIC about the change in their quality of life, physical status, and emotional status after 1 week. To assess the test-retest reliability of the Japanese version of the PRO-CTCAE, 14 items were re-assessed on Day 2 in Cohort A. Data were collected using paper and pencil at two facilities, Juntendo University Nerima Hospital and Toshiba General Hospital, and using the electronic PRO (ePRO) platform at two facilities, Tokyo Medical University Hospital and Saitama Medical University Saitama Medical Center. Mode equivalence between paper and ePRO of the PRO-CTCAE has been shown to be good [30, 31]. The hospital pharmacist obtained patients’ informed consent and administered the questionnaire. The number of samples was not calculated statistically and was set to 100 for cohort A and 80 for cohort B, based on the feasibility of 1 year, which was the test period of this study.

### Statistical analyses

Descriptive statistics were used to summarize baseline patient characteristics. Test-retest reliability was assessed using the intra-class correlation coefficient (ICC). The threshold value of the ICC is a matter of controversy. While some think it should be 0.7 or above, others consider such an absolute threshold to be too prescriptive [32]. Given that this study includes patients undergoing chemotherapy, who are prone to change of condition, we adopted the threshold value defined by Cicchetti and colleagues [33]. The threshold value of the ICC is considered poor when the ICC is less than 0.4, fair when it is 0.4 to 0.59, good when it is 0.6 to 0.74, and excellent when it is greater than or equal to 0.75. To assess construct validity, Pearson correlation coefficients were computed between each item on the Japanese version of the PRO-CTCAE and each item score on the EORTC QLQ-C30. In this construct validation, we checked that the PRO-CTCAE and EORTC correlated with scores on corresponding items and not with non-corresponding items. Responsiveness was assessed by comparing changes in the Japanese version of the PRO-CTCAE item scores, as a numerical value ranging from 1 to 4 or 5, from the first to second visit of Cohort B. Standardized response means (SRMs), which were calculated by dividing the mean score change by the standard deviation of the score change, were computed for each GIC category, and trends were investigated by the Jonckheere–Terpstra trend test. Regarding the discordance between the CTCAE (assessed by the clinician) and the Japanese version of the PRO-CTCAE (assessed by patients), the proportion of patients who reported adverse events on the Japanese version of the PRO-CTCAE to that of patients evaluated as having no adverse events by the CTCAE was examined. Statistical analyses were performed using JMP PRO (Version 13.0.0, SAS Institute Inc., Cary, NC, USA) and SPSS 24 (IBM SPSS Statistics 24, IBM Corporation, Somers, NY).

## Results

### Participants

**Fig. 1:**
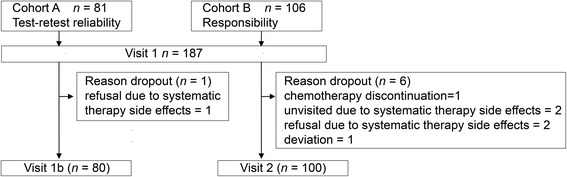
Flow of experimental protocols

A total of 187 patients were recruited from March 2015 to March 2016. One patient in Cohort A and six patients in Cohort B dropped out before Visit 2, resulting in 80 patients included in analyses for Cohort A and 100 patients for Cohort B (Fig. 1). The mean (*SD*) age of the total sample was 62.4 (10.7) years [63.9 (10.6) years for Cohort A, and 61.2 (10.8) years for Cohort B; Table 1]. Breast and gastrointestinal cancer were common in both cohorts. More lung cancer patients were enrolled in Cohort A as compared to Cohort B, whereas more patients with head and neck cancer were enrolled in Cohort B. A majority of the patients received chemotherapy without molecular target medicine as curative treatment. Ninety percent or more of patients demonstrated ECOG PS scores of 0 or 1, and scores of 80 or more on the KPS. In all, 164 patients (87.7%) responded to the questionnaire via electronic tablet and 23 (12.3%) by paper; thus, the overall response rate was 100%. Further, all participants reported at least one symptom at Visit 1. Of the 39 items surveyed in this study, patients answered that they exhibited an average of 16 items (at least 1 item, maximum of 36 items per patient).

**Table 1:** Participant characteristics

		Total (*n* = 187)		Cohort A (test-retest) (*n* = 80)		Cohort B (responsiveness) (*n* = 100)	
Age							
	Total	62.4	10.7	63.9	10.6	61.2	10.8
	Male	64.5	9.3	65.7	9.2	63.4	9.3
	Female	59.6	11.8	61.5	12.0	58.1	12.0
		*n*	%	*n*	%	*n*	%
Total							
	Male	107	57.2	45	56.3	58	58.0
	Female	80	42.8	35	43.8	42	42.0
Cancer diagnosis							
	Breast	25	13.4	12	15.0	12	12.0
	Gastrointestinal	91	48.7	37	46.3	50	50.0
	Gynecological	17	9.1	4	5.0	12	12.0
	Lung	32	17.1	27	33.8	4	4.0
	Head and Neck	20	10.7	0	0	20	20.0
	Other	2	1.0	0	0	2	2.0
Current therapy							
	Chemotherapy	185	98.9	80	100.0	98	98.0
	With radiation	30	16.0	9	11.3	21	21.0
Type of therapy							
	Adjuvant	61	32.6	26	32.5	33	33.0
	Palliative	126	67.4	54	67.5	67	67.0
Molecular target drug use							
	Yes	46	24.6	22	27.5	22	22.0
	No	141	75.4	58	72.5	78	78.0
KPS							
	100	44	23.5	31	38.8	12	12.0
	90	124	66.3	43	53.8	75	75.0
	80	15	8.0	2	2.5	13	13.0
	< 70	4	2.1	4	5.0	0	0.0
ECOG PS							
	0	74	39.6	37	46.3	34	34.0
	1	108	57.8	39	48.8	65	65.0
	2	4	2.1	3	3.8	1	1.0
	3	1	0.5	1	1.3	0	0.0
Marital status							
	Married	135	72.2	57	71.3	72	72.0
	Unmarried	25	13.4	10	12.5	14	14.0
	Divorced	11	5.9	5	6.3	6	6.0
	Bereaved	16	8.6	8	10.0	8	8.0
Education							
	High	54	28.9	27	33.8	25	25.0
	Middle	119	63.6	48	60.0	66	66.0
	Low	14	7.5	5	6.3	9	9.0
Employment status							
	Full-time	57	30.5	26	32.5	29	29.0
	Part-time	13	7.0	5	6.3	8	8.0
	Unemployed or retired	58	31.0	24	30.0	31	31.0
	Homemaker	35	18.7	18	22.5	17	17.0
	Others	24	12.8	7	8.8	15	15.0
Household							
	With family	156	83.4	63	78.8	87	87.0
	Alone	28	15.0	16	20.0	11	11.0
	Institution	3	1.6	1	1.3	2	2.0

### Discordance between clinicians and patients

**Fig. 2:**
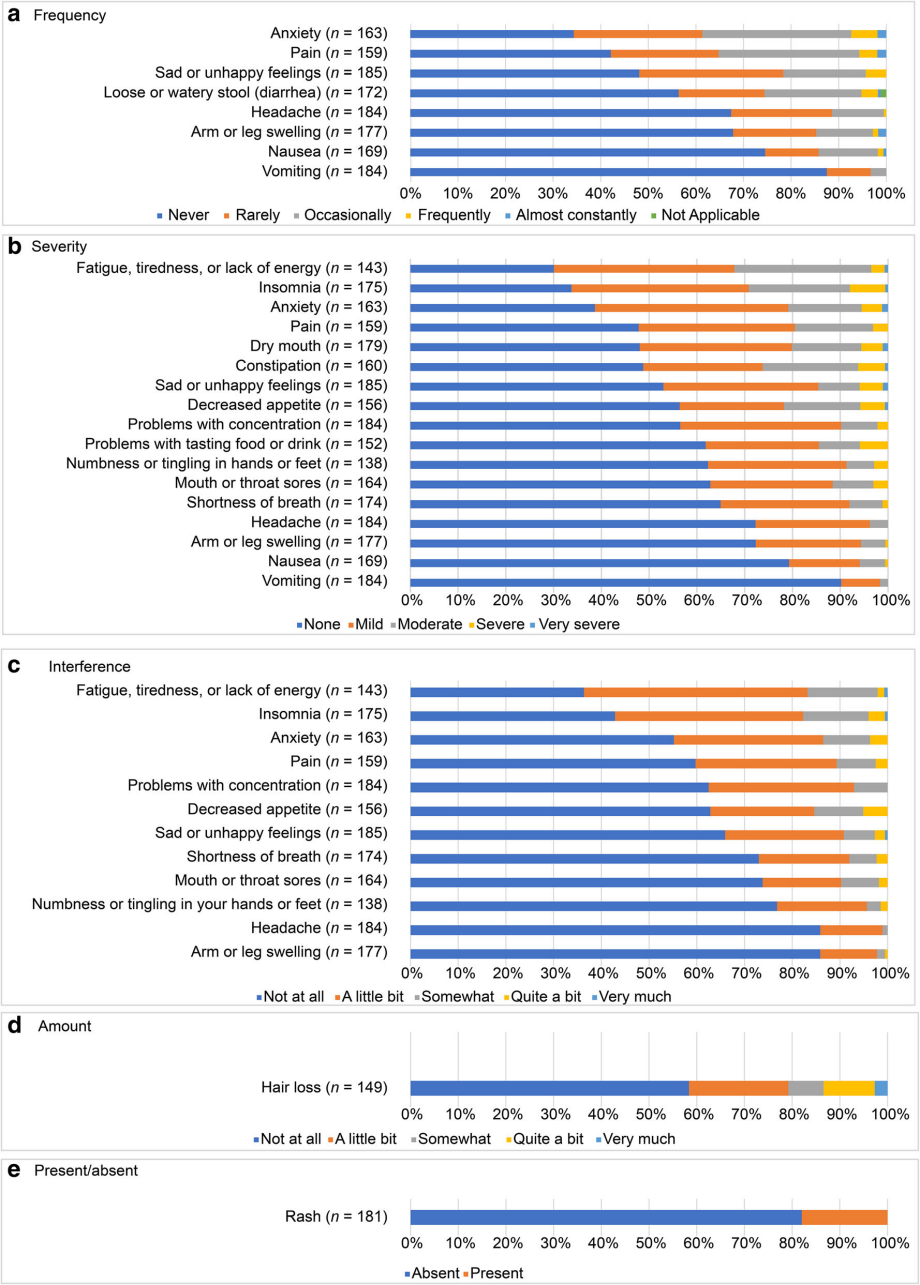
Response of PRO-CTCAE in patients assessed by the CTCAE as having no adverse events. (a: Frequency, b: Severity, c: Interference, d: Amount, and e: Present/absent.)

Self-symptom assessment using the Japanese PRO-CTCAE in patients evaluated as non-graded by the clinician is shown in Fig. 2. Regarding frequency and severity, the proportion of patients who self-reported having no adverse events to that of patients rated by the clinician as having no adverse events was high for “vomiting” (87.5% for frequency, 90.2% for severity), “nausea” (74.6% for frequency, 79.3% for severity) and “swelling in the arms or legs” (67.8% for frequency, 72.3% for severity), but low for “anxiety” (34.3% for frequency, 38.7% for severity), “pain” (42.1% for frequency, 47.8% for severity) and “sad or unhappy feelings” (48.1% for frequency, 53.0% for severity).

### Reliability

The ICCs between responses on the Japanese version of the PRO-CTCAE items on Day 1 and 2 are shown in Table 2. The mean (95% CI) ICC for the whole scale (*n* = 28) was 0.63 (0.59–0.68). The mean ICCs for the attributes of frequency (*n* = 6), severity (*n* = 13), and interference (*n* = 9) were 0.67 (0.57–0.77), 0.62 (0.54–0.70), and 0.63 (0.56–0.71), respectively. Of the 28 items from the 14 symptoms evaluated, the ICC for 16 items (57.1%) exceeded 0.6, and only one item (vomiting, severity) had an ICC of less than 0.4.

**Table 2:** Test-retest reliability of selected PRO-CTCAE items (*n* = 80)

PRO-CTCAE items	Attributes	ICC	95% CI	
			Lower	Upper
Anxiety				
	Frequency	0.56	0.39	0.69
	Severity	0.55	0.38	0.69
	Interference	0.64	0.49	0.75
Constipation				
	Severity	0.63	0.48	0.75
Decreased appetite				
	Severity	0.48	0.29	0.63
	Interference	0.58	0.41	0.70
Dry mouth				
	Severity	0.74	0.62	0.82
Fatigue, tiredness, or lack of energy				
	Severity	0.57	0.41	0.70
	Interference	0.65	0.51	0.76
Insomnia				
	Severity	0.52	0.34	0.66
	Interference	0.59	0.42	0.71
Loose or watery stools (diarrhea)				
	Frequency	0.78	0.67	0.85
Mouth or throat sores				
	Severity	0.58	0.41	0.71
	Interference	0.65	0.50	0.76
Nausea				
	Frequency	0.70	0.56	0.79
	Severity	0.55	0.38	0.69
Numbness or tingling in hands or feet				
	Severity	0.76	0.65	0.84
	Interference	0.46	0.27	0.61
Pain				
	Frequency	0.74	0.62	0.82
	Severity	0.76	0.65	0.84
	Interference	0.77	0.67	0.85
Sad or unhappy feelings				
	Frequency	0.69	0.56	0.79
	Severity	0.80	0.70	0.87
	Interference	0.77	0.66	0.84
Shortness of breath				
	Severity	0.75	0.64	0.83
	Interference	0.61	0.45	0.73
Vomiting				
	Frequency	0.55	0.38	0.69
	Severity	0.35	0.14	0.53

### Validity

The items with an absolute value of Pearson correlation coefficient of 0.4 or more between the function score on the EORTC QLQ-C30 and the items on the Japanese version of the PRO-CTCAE are shown in Table 3. The GHS/QOL correlated with “decreased appetite” (interference: Pearson *r* = −0.43, 95% CI: -0.54 to −0.31) and “fatigue, tiredness, or lack of energy” (severity: Pearson *r* = −0.45, 95% CI: -0.55 to −0.32; interference: Pearson *r* = −0.51, 95% CI: -0.61 to −0.40). There was a modest correlation between the physical functioning score and the Japanese version of the PRO-CTCAE items “fatigue, tiredness, or lack of energy” (severity: Pearson *r* = −0.43, 95% CI: -0.58 to −0.30; interference: Pearson *r* = −0.49, 95% CI: -0.59 to −0.38) and “shortness of breath” (severity: Pearson *r* = −0.52, 95% CI: -0.62 to −0.40; interference: Pearson *r* = −0.51, 95% CI: -0.60 to −0.39). Similarly, “anxiety,” “sad or unhappy feelings,” and “problems with concentration” were correlated with the emotional functioning score. There was a correlation between the cognitive functioning score and the “problem with concentration” score (severity: Pearson *r* = −0.52, 95% CI: -0.62 to −0.40; interference: Pearson *r* = −0.47, 95% CI: -0.57 to −0.35). Table 4 shows the correlations between the symptom scores on the EORTC QLQ-C30 and the Japanese version of the PRO-CTCAE. As indicated in bold in Table 4, the correlation coefficients for the corresponding items on the EORTC QLQL-C30 and the Japanese version of PRO-CTCAE were high, while those for the items that did not correspond tended to be low.

**Table 3:** Pearson correlation coefficients between the function score on the EORTC QLQ-C30 and items of the PRO-CTCAE

	Pearson correlation	95% CI	
		Lower	Upper
GHS/QOL			
Decreased appetite, interference	−0.43	−0.54	−0.31
Fatigue, tiredness, or lack of energy, severity	−0.45	−0.55	−0.32
Fatigue, tiredness, or lack of energy, interference	−0.51	−0.61	−0.40
Physical functioning			
Fatigue, tiredness, or lack of energy, severity	−0.43	−0.54	−0.30
Fatigue, tiredness, or lack of energy, interference	−0.49	−0.59	−0.38
Shortness of breath, severity	−0.52	−0.62	−0.40
Shortness of breath, interference	−0.51	−0.60	−0.39
Role functioning			
Decreased appetite, severity	−0.42	−0.53	−0.29
Decreased appetite, interference	−0.49	−0.59	−0.38
Fatigue, tiredness, or lack of energy, severity	−0.49	−0.60	−0.38
Fatigue, tiredness, or lack of energy, interference	−0.63	−0.71	−0.53
Nausea, severity	−0.41	−0.52	−0.28
Shortness of breath, severity	−0.40	−0.52	−0.27
Shortness of breath, interference	−0.43	−0.54	−0.31
Emotional functioning			
Anxiety, frequency	−0.53	−0.63	−0.42
Anxiety, severity	−0.60	−0.68	−0.50
Anxiety, interference	−0.48	−0.58	−0.36
Sad or unhappy feelings, frequency	−0.61	−0.69	−0.51
Sad or unhappy feelings, severity	−0.66	−0.73	−0.57
Sad or unhappy feelings, interference	−0.60	−0.68	−0.50
Problems with concentration, severity	−0.41	−0.52	−0.28
Problems with concentration, interference	−0.46	−0.57	−0.34
Cognitive functioning			
Problems with concentration, severity	−0.52	−0.62	−0.40
Problems with concentration, interference	−0.47	−0.57	−0.35

**Table 4:** Pearson correlation coefficients between symptom assessment by the PRO-CTCAE versus the EORTC QLQ-C30

	EORTC QLQ-C30							
PRO-CTCAE items at Visit 1	Fatigue	Nausea/Vomiting	Pain	Dyspnea	Insomnia	Appetite loss	Constipation	Diarrhea
Fatigue, tiredness, or lack of energy, Severity	**0.56**	0.35	0.28	0.20	0.21	0.48	0.11	0.18
Fatigue, tiredness, or lack of energy, Interference	**0.59**	0.39	0.32	0.27	0.20	0.49	0.02	0.13
Nausea, Frequency	0.32	**0.76**	0.25	0.15	0.17	0.36	0.22	0.24
Nausea, Severity	0.33	**0.77**	0.27	0.09	0.15	0.38	0.16	0.27
Vomiting, Frequency	0.15	**0.64**	0.13	0.11	0.10	0.20	0.16	0.18
Vomiting, Severity	0.16	**0.63**	0.16	0.07	0.14	0.18	0.09	0.18
Pain, Frequency	0.21	0.10	**0.65**	0.04	0.25	0.14	0.12	0.06
Pain, Severity	0.27	0.11	**0.70**	0.09	0.31	0.16	0.07	0.10
Pain, Interference	0.32	0.11	**0.69**	0.07	0.30	0.26	0.06	0.14
Shortness of breath, Severity	0.39	0.10	0.11	**0.66**	0.12	0.13	0.03	−0.04
Shortness of breath, Interference	0.42	0.10	0.23	**0.64**	0.19	0.10	0.05	−0.03
Insomnia, Severity	0.26	0.17	0.24	0.16	**0.62**	0.22	0.16	0.27
Insomnia, Interference	0.33	0.16	0.28	0.21	**0.67**	0.29	0.17	0.24
Decreased appetite, Severity	0.45	0.51	0.30	0.13	0.28	**0.71**	0.14	0.30
Decreased appetite, Interference	0.49	0.48	0.38	0.14	0.34	**0.69**	0.11	0.32
Constipation, Severity	0.02	0.12	0.10	−0.04	0.16	0.10	**0.65**	0.06
Loose or watery stools (diarrhea), frequency	0.14	0.15	0.12	0.09	0.20	0.21	−0.01	**0.71**

Bold: Corresponding item between the ELRTC QLQ-C30 and PRO-CTCAE

**Fig. 3:**
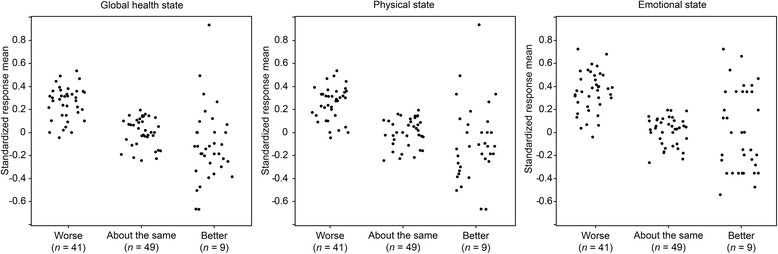
Standardized response means of 39 PRO-CTCAE items by patient-reported global impression of change (Global health, physical, and emotioinal state). Significant trends were observed in each status. (Jonckheere–Terpstra test, *ps* < 0.001)

The SRMs of the Japanese version of the PRO-CTCAE are shown in Fig. 3  (see Appendix 2). Concerning the global health state, the means (95% CI) of the SRMs according to the three GIC categories were *worse* [−0.002 (0.058)], *almost the same* [−0.019 (0.020)], and *better* [−0.257 (0.022)], respectively. Regarding physical and emotional state, the means (95% CI) were as follows: physical state: *worse,* 0.257 (0.209–0.304); *almost the same*, 0.005 (−0.040–0.047); and *better* − 0.101 (−0.210–0.007); emotional state: *worse*, 0.353 (0.295–0.412); *almost the same*, 0.012 (−0.030–0.052); and *better*, 0.004 (−0.120–0.124); for the three GIC categories, respectively. In all states of the GIC, significant trends (namely dose dependent relationships) were observed (Jonckheere–Terpstra test, *p*s < 0.001).

## Discussion

This study aimed to investigate the discordance of adverse event assessment between clinicians and patients using the CTCAE and the Japanese version of the PRO-CTCAE, as well as to survey the psychometric properties of the Japanese version of the PRO-CTCAE. Results indicate that there is a discrepancy in evaluation between clinicians and patients, depending on the adverse events, and that the validity and reliability of the Japanese version of the PRO-CTCAE is acceptable.

First, our findings indicated that the CTCAE grades and the responses to the PRO-CTCAE did not identically correspond. Thus, we evaluated the discordance of adverse event assessment in this study, via patient self-assessment using the PRO-CTCAE, in patients assessed by medical staff as having no adverse events, via the CTCAE. Underestimation of adverse events by clinicians have been previously reported using the CTCAE and/or EORTC QLQ-C30 [3, 4, 7]. These reports have consistently shown an underestimation of fatigue, pain, and constipation by clinicians, a trend mirrored in the current study. Additionally, our results also showed that anxiety was underestimated. The Japanese version of the PRO-CTCAE was shown to have properties similar to trends demonstrated in previous studies utilizing this measure in other countries. In Japan, there is a dearth of research examining the underestimation of adverse events. Okamoto has proposed that patients believe that doctors do not actively engage in listening to their concerns, while doctors tend to believe that patients are not willing to voice their opinions [34]. Within the unique context of Japanese physician-patient relationships, the Japanese version of the PRO-CTCAE is considered likely to affect the underestimation of adverse events, resulting in a more appropriate assessment. Finally, although this study revealed the existence of underestimation of adverse events in Japan, the sample utilized contained multiple different cancer types and treatment regimens. Further studies will be necessary to clarify discordance of particular treatments and/or cancer-type-specific adverse event assessments.

In addition, although almost all of the ICCs in evaluating the reproducibility of the Japanese version of the PRO-CTCAE were fair or good, the ICCs for “vomiting, severity (0.35)” and “decreased appetite, severity (0.48)” were relatively low. In almost all patients, these data evaluated the degree of agreement before and after the start of chemotherapy, and this timing is often defined as the acute phase in clinical trials to assess the effectiveness of antiemetic agents. A recent study in a Japanese population showed that the Complete Response (no vomiting/retching and no rescue medication) and Complete Control (no vomiting/retching, no rescue medication, and no more than mild nausea) of nausea and vomiting in the acute phase of highly emetogenic chemotherapy is approximately 75 to 90%, and the Total Control (no vomiting/retching, no rescue medication, and no nausea) is between 80 and 87% [35–37]. As can be seen from these data, it is well known that chemotherapy causes nausea and vomiting in the acute phase, much of which can be alleviated by antiemetic agents, although not completely controlled. The low ICCs found for some items may reflect symptomatic changes in the acute phase before and after chemotherapy.

Within the attributes of the same item, there was not much difference between ICC values. However, for “Numbness or tingling in hands or feet” there was a relatively large difference between severity and interference. A similar tendency exists in the original version [16]. In many cases, chemotherapy-induced neuropathy becomes reversible to irreversible in a dose-dependent fashion. However, because the ICC for severity in “Numbness or tingling in hands or feet” was shown to be good in the current study, it is considered that the low value of the interference ICC is not due to a change in this symptom. Recently, a multidimensional scale for assessing chemotherapy-induced peripheral neuropathy (CIPN), called the EORTC QLQ CIPN20, has been developed [38]. Wolf and colleagues conducted a study using this scale and argued that the evaluation of numbness of hands and feet should be strictly distinguished [39]. They revealed that neuropathic symptoms, such as numbness or tingling, tend to appear more strongly in lower extremities than upper extremities. Therefore, it is considered that a change in the influence on daily life, especially in the feet, caused by hospitalization owing to the start of chemotherapy, is one of the reasons for the deviation in the ICC for “Numbness or tingling in hands or feet.” It should be noted when interpreting the change in this item however, that the data currently available are preliminary.

This study has several limitations. First, the psychometric properties of the Japanese version of the PRO-CTCAE revealed in this study were investigated in a subset of core items that were specifically selected as a result of previous research. Furthermore, although it is the same as the original validation study, test-retest reliability was examined with only 14 items on the Japanese PRO-CTCAE. Although it seems to be sufficient for interpretation, it should be noted that these findings are not the result of investigating all items of the Japanese version of the PRO-CTCAE. Secondly, we were unable to recruit patients with poor performance status. Although Dueck provided known group validity for these individuals during development of the original version [16], the relationship between this group and the Japanese version of the PRO-CTCAE was not clarified. Finally, the current study focused on underestimation of adverse events but did not assess overestimation. Future research should consider whether there is an overestimation of adverse events that leads to overtreatment.

## Conclusions

This study revealed that the discordance between clinician and patient assessments of adverse events was similar to that of previous reports, and that the Japanese version of the PRO-CTCAE demonstrated acceptable reliability and validity for common and clinically important symptoms. It is expected that the Japanese version of the PRO-CTCAE will be applied to patient-centered evaluation of adverse events in future clinical trials in Japan.

## Appendix 1

**Table 5:** Japanese version of the PRO-CTCAE items used in the current study

	Visit 1 (Distribution/Criterion validity)	Visit 1b (Reliability)	Visit 2 (Responsiveness)
Anxiety	○	○	○
Constipation	○	○	○
Decreased appetite	○	○	○
Dry mouth	○	○	○
Fatigue, tiredness, or lack of energy	○	○	○
Insomnia	○	○	○
Loose or watery stools (diarrhea)	○	○	○
Mouth or throat sores	○	○	○
Nausea	○	○	○
Numbness or tingling in hands or feet	○	○	○
Pain	○	○	○
Sad or unhappy feelings	○	○	○
Shortness of breath	○	○	○
Vomiting	○	○	○
Arm or leg swelling	○		○
Hair loss	○		○
Headache	○		○
Problems with concentration	○		○
Problems with tasting food or drink	○		○
Rash	○		

## Appendix 2

**Table 6:** Standardized response mean in Cohort B (*n* = 100)

	Overall Quality of Life			Physical State			Emotional State		
	Improved	No change	Worse	Improved	No change	Worse	Improved	No change	Worse
Anxiety									
Frequency	−0.11	−0.06	−0.30	0.00	−0.02	−0.37	0.35	0.05	−0.72
Severity	0.15	0.02	−0.25	0.27	0.10	−0.36	0.35	0.10	−0.53
Interference	0.00	−0.17	−0.21	0.12	−0.06	−0.30	0.54	−0.05	−0.50
Constipation									
Severity	−0.13	0.10	−0.36	0.00	0.19	−0.47	−0.13	−0.05	−0.24
Decreased appetite									
Severity	0.15	−0.11	−0.29	0.38	0.02	−0.36	0.15	−0.14	−0.31
Interference	0.24	−0.15	−0.25	0.67	0.06	−0.39	0.24	−0.09	−0.39
Dry mouth									
Severity	0.36	0.19	−0.23	0.18	0.22	−0.27	0.35	−0.02	−0.04
Fatigue, tiredness, or lack of energy									
Severity	−0.28	−0.10	−0.26	−0.10	−0.14	−0.23	0.20	−0.10	−0.37
Interference	0.00	0.00	−0.47	0.12	0.02	−0.44	0.35	−0.07	−0.58
Insomnia									
Severity	−0.23	−0.05	−0.10	−0.07	−0.09	−0.10	0.00	−0.06	−0.16
Interference	0.00	−0.18	0.02	0.14	−0.15	0.00	0.20	0.00	−0.22
Loose or watery stools (diarrhea)									
Frequency	0.06	−0.14	0.02	−0.49	−0.07	0.00	0.47	−0.12	−0.07
Mouth or throat sores									
Severity	0.00	0.20	−0.21	0.12	0.17	−0.20	0.00	0.17	−0.26
Interference	−0.35	0.03	−0.38	−0.12	−0.09	−0.27	−0.35	−0.03	−0.36
Nausea									
Frequency	−0.35	−0.20	−0.39	−0.18	−0.13	−0.49	−0.35	−0.19	−0.50
Severity	−0.54	−0.24	−0.32	−0.27		−0.33	−0.54	−0.19	−0.42
Numbness or tingling in your hands or feet									
Severity	–	−0.04	0.03	–	0.03	−0.05	−0.35	0.00	0.04
Interference	−0.35	−0.17	−0.17	−0.33	−0.16	−0.22		−0.14	−0.30
Pain									
Frequency	0.77	0.14	−0.02	0.39	0.24	−0.02	0.23	0.23	−0.15
Severity	1.20	0.06	−0.10	0.30	0.16	−0.10	0.00	0.10	−0.68
Interference	0.35	0.00	−0.13	0.00	0.11	−0.17	−0.20	0.14	−0.30
Sad or unhappy feeling									
Frequency	0.00	−0.20	−0.34	−0.33	−0.19	−0.32	0.28	−0.11	−0.59
Severity	0.20	0.23	−0.19	0.18	0.16	−0.15	0.35	0.26	−0.32
Interference	0.20	0.20	−0.29	0.18	0.23	−0.32	0.35	0.17	−0.39
Shortness of breath									
Severity	0.36	0.09	−0.45	0.50	0.04	−0.38	0.35	−0.10	−0.38
Interference	0.36	−0.08	−0.54	0.47	−0.04	−0.53	0.35	−0.19	−0.53
Vomiting									
Frequency	−0.36	0.05	−0.27	0.33	−0.14	−0.22	−0.35	−0.03	−0.46
Severity	–	−0.06	−0.32	–	−0.15	−0.24	–	−0.04	−0.54
Arm or leg swelling									
Frequency	0.00	−0.07	−0.30	0.20	−0.05	−0.29	−0.47	0.05	−0.40
Severity	0.00	0.05	−0.35	0.11	0.00	−0.31	−0.41	0.18	−0.51
Interference	0.00	0.00	−0.31	0.00	0.00	−0.30	−0.20	0.00	−0.33
Hair loss									
Amount	−0.32	0.17	−0.08	−0.93	0.03	0.05	−0.13	0.14	−0.19
Headache									
Frequency	0.00	−0.02	−0.22	0.36	−0.10	−0.09	−0.66	−0.05	−0.06
Severity	−0.20	0.02	−0.30	0.10	−0.07	−0.15	−0.72	−0.04	−0.13
Interference	−0.20	−0.08	−0.40	0.66	−0.14	−0.31	−0.35	0.12	−0.39
Problems with concentration									
Severity	−0.51	−0.08	−0.35	0.25	−0.12	−0.36	−0.41	−0.13	−0.35
Interference	−0.20	−0.11	−0.32	0.23	−0.11	−0.33	0.20	−0.10	−0.46
Problems with tasting food or drink									
Severity	−0.36	0.02	−0.36	0.18	0.03	−0.35	0.15	−0.12	−0.33

## Abbreviations

CIPN: Chemotherapy-induced peripheral neuropathy
CTCAE: Common Terminology Criteria for Adverse Events
CTEP: Cancer Therapy Evaluation Program
ECOG PS: Eastern Cooperative Oncology Group Performance Status scale
EORTC QLQ CIPN20: European Organisation of Research and Treatment of Cancer Quality of Life Chemotherapy-Induced peripheral neuropathy
EORTC QLQ-C30: European Organisation of Research and Treatment of Cancer Quality of Life Questionnaire Core 30
ePRO: Electronic patient-reported outcomes
GHS/QOL: Global Health Status/Quality of Life
GIC: Global impression of change
ICC: Intra-class correlation coefficient
JCOG: Japan Cooperative Oncology Group
KPS: Karnofsky Performance Status
MedDRA: Medical Dictionary for Regulatory Activities
NCI: National Cancer Institute
PRO: Patient-reported outcomes
PRO-CTCAE: Patient-Reported Outcomes version of the Common Terminology Criteria for Adverse Events
SRM: Standardized response means
